# A review of heat stress in chickens. Part II: Insights into protein and energy utilization and feeding

**DOI:** 10.3389/fphys.2022.943612

**Published:** 2022-08-08

**Authors:** Jean-Rémi Teyssier, Giorgio Brugaletta, Federico Sirri, Sami Dridi, Samuel J. Rochell

**Affiliations:** ^1^ Center of Excellence for Poultry Science, University of Arkansas, Fayetteville, AR, United States; ^2^ Department of Agricultural and Food Sciences, Alma Mater Studiorum—University of Bologna, Bologna, Italy

**Keywords:** heat stress, chicken, feed intake, digestibility, feeding strategies, amino acids, energy, broiler

## Abstract

With the growing global demand for animal protein and rising temperatures caused by climate change, heat stress (HS) is one of the main emerging environmental challenges for the poultry industry. Commercially-reared birds are particularly sensitive to hot temperatures, so adopting production systems that mitigate the adverse effects of HS on bird performance is essential and requires a holistic approach. Feeding and nutrition can play important roles in limiting the heat load on birds; therefore, this review aims to describe the effects of HS on feed intake (FI) and nutrient digestibility and to highlight feeding strategies and nutritional solutions to potentially mitigate some of the deleterious effects of HS on broiler chickens. The reduction of FI is one of the main behavioral changes induced by hot temperatures as birds attempt to limit heat production associated with the digestion, absorption, and metabolism of nutrients. Although the intensity and length of the heat period influences the type and magnitude of responses, reduced FI explains most of the performance degradation observed in HS broilers, while reduced nutrient digestibility appears to only explain a small proportion of impaired feed efficiency following HS. Targeted feeding strategies, including feed restriction and withdrawal, dual feeding, and wet feeding, have showed some promising results under hot temperatures, but these can be difficult to implement in intensive rearing systems. Concerning diet composition, feeding increased nutrient and energy diets can potentially compensate for decreased FI during HS. Indeed, high energy and high crude protein diets have both been shown to improve bird performance under HS conditions. Specifically, positive results may be obtained with increased added fat concentrations since lipids have a lower thermogenic effect compared to proteins and carbohydrates. Moreover, increased supplementation of some essential amino acids can help support increased amino acid requirements for maintenance functions caused by HS. Further research to better characterize and advance these nutritional strategies will help establish economically viable solutions to enhance productivity, health, welfare, and meat quality of broilers facing HS.

## Introduction

The poultry industry continues to play a critical role in meeting the growing demand for animal protein. The global production of chicken and turkey meat has doubled over the last 20 years, reaching 125.5 million tons in 2020 (FAO, 2022). This accounts for approximately 37% of global meat production, while poultry meat only represented 29% in the early 2000s. With the increasing global population projected to rise from 7.8 to 9.9 billion in 2050 (PRB, 2020) and better access to animal products in developing areas, it is predicted that animal-based food demand will grow by nearly 70% in the same timeline (Searchinger et al., 2019). Meanwhile, climate change represents one of the major concerns for livestock production in the coming decades. Some reports indicate that industrialized farming systems may lose 25% of their animal production, and this scenario may be worse for some tropical regions where extensive farming systems are more abundant (Nardone et al., 2010). Emerging estimates by the Intergovernmental Panel on Climate Change emphasized that global warming of more than 2°C will occur during the 21st century unless large reductions in CO_2_ and other greenhouse gas emissions are acheived soon (IPCC, 2021). Also, the authors indicated that climate change is already and will continue increasing the frequency and intensity of extreme weather events like hot temperature waves. Therefore, the poultry industry needs to continue adopting technologies and practices that reduce its impact on the environment, but it should also adopt production systems that are resilient in the face of rising global temperatures.

Modern broiler chickens are particularly sensitive to hot temperatures due to their rapid growth rates resulting from genetic selection to enhance production efficiency, as well as from limitations in heat dissipation caused by feathering, an absence of sweat glands, and relatively high stocking densities in intensive commercial rearing facilities (Lara and Rostagno, 2013; Emami et al., 2020). Heat stress (HS) occurs when the amount of heat produced by an animal surpasses its capacity to dissipate the heat to the surrounding environment. When the environmental temperature rises above the thermoneutral zone, birds typically reduce their physical activity and feed intake (FI) to limit heat production (HP), as well as increase their panting and water consumption to favor heat loss by evaporation (Renaudeau et al., 2012). Indeed, elevated temperatues trigger important physiologic and metabolic changes as described in Part I of this review (Brugaletta et al., 2022), and chronic HS exposure results in significant losses in bird performance, negatively affects welfare, challenges food safety, and reduces the overall economic efficiency of poultry production (Lara and Rostagno, 2013; Pawar et al., 2016). Consequently, HS has been estimated to cause annual economic losses of $128 to $165 million for the United States poultry industry (St-Pierre et al., 2003), but these figures probably underestimate current and future losses due to the growth of the poultry industry over the last decade and the worsening of climate change predictions.

Mitigating the adverse effects caused by hot temperatures in poultry productions requires a holistic and multi-factorial approach. Housing (Oloyo, 2018), management practices (Saeed et al., 2019), genetic selection (Kumar et al., 2021), and feeding and nutrition (Syafwan et al., 2011; Fouad et al., 2016; Sugiharto et al., 2017; Wasti et al., 2020; Abdel-Moneim et al., 2021; Chowdhury et al., 2021) can all provide some benefit to birds under HS conditions and have been the topics of several recent global reviews (Lin et al., 2006b; Nawab et al., 2018; Goel et al., 2021; Vandana et al., 2021). This review aims to assess the effects of HS on FI and nutrient digestibility, as well as to evaluate different feeding strategies and nutritional solutions to mitigate some of the adverse effects of HS on poultry. Effects on broiler chickens will be emphasized, though research with other types of poultry will be discussed where relevant. Further, this review will focus on macro-nutritional solutions as carbohydrates, fat, and proteins are the main source of energy, and their oxidation results in HP (Costa-Pinto and Gantner, 2020), which needs to be limited under HS.

## Impact of heat stress on feed intake regulation and nutrient digestibility

Nearly all studies that have investigated the effects of HS in poultry have observed reductions in FI of heat-stressed birds compared with those in thermoneutral conditions, including meta-analyses conducted in broilers (Liu et al., 2020) and laying hens (Mignon-Grasteau et al., 2015). This reduction of FI observed under HS conditions reduces endogenous HP associated with digestion, absorption, and metabolism of nutrients (Lara and Rostagno, 2013). However, the magnitude of the FI reduction depends on several parameters related to the characteristics of the HS model imposed on the birds, and this can complicate comparisons among studies. Temperature, length and cyclicality of the heat period, and age of the birds at the beginning and the end of the HS period are all potential factors that can influence the intensity of the FI reduction. Many studies have used a constant HS model with high temperatures applied over a long period of time (Baziz et al., 1996; Geraert et al., 1996; Bonnet et al., 1997; Faria Filho et al., 2007). However, more recent studies have employed cyclic HS models combining higher temperatures during the day and lower temperatures during the night which may better simulate field conditions in temperate areas of the world (De Souza et al., 2016; Flees et al., 2017; Greene et al., 2021). When compared within the same experiment, cyclic HS decreased FI by 15% on average, while constant HS resulted in higher reductions ranging from 25% to 45% (De Souza et al., 2016; Awad et al., 2018; Teyssier et al., 2022). Therefore, cyclic HS resulted in a 1.5% reduction in FI per degree Celsius, while the values obtained under constant HS corroborate the expected response proposed by Baziz et al. (1996) of about a 3.5% reduction in FI per degree Celsius increase between 22°C and 35°C.

Interestingly, the reduction of growth observed under HS is greater than expected due to the reduced FI alone, leading to a lower feed efficiency (Renaudeau et al., 2012). The use of pair-feeding techniques, where birds under thermoneutral conditions are fed the same amount of feed consumed by heat-stressed birds, have shown that the reduction in growth due to decreased FI ranges between 60% and 99% (Geraert et al., 1996; Bonnet et al., 1997; Garriga et al., 2006; Lu et al., 2007; Zuo et al., 2015; De Souza et al., 2016; Zeferino et al., 2016; De Antonio et al., 2017; Emami et al., 2021; Ma et al., 2021; Teyssier et al., 2022). Therefore, the lower FI is the main factor explaining impaired performance of chickens observed under HS, with the remainder of the growth reduction attributable to impaired digestibility or physiological and metabolic changes that influence feed efficiency (Dale and Fuller, 1980; Geraert et al., 1996; Renaudeau et al., 2012).

Several studies have reported reduced dry matter (DM) digestibility in quails (Orhan et al., 2020) and laying hens (Kim et al., 2020) under HS conditions. In broilers, Bonnet et al. (1997) and De Souza et al. (2016) observed decreases of 1.6% and 3.9% in DM digestibility under constant HS. However, other studies have reported no DM digestibility losses due to HS (Faria Filho et al., 2007; Attia et al., 2016, 2017). At the nutrient level, even though no change in crude protein (CP) digestibility were observed by several authors (Faria Filho et al., 2007; Habashy et al., 2017b; Kim et al., 2020), numerous studies have reported decreases in CP or nitrogen digestibility ranging between 1.5% and 10% under hot temperatures (Zuprizal et al., 1993; Bonnet et al., 1997; Soleimani et al., 2010; Attia et al., 2016, 2017; De Souza et al., 2016; Orhan et al., 2020). The detrimental effect of HS has also been measured on amino acid (AA) digestibility. Wallis and Balnave (1984) observed a slight decrease in the digestibility for Thr, Ala, Met, Ile, and Leu, with greater impacts in male than in female birds. Standardized and apparent digestibility values of several AA (i.e., Arg, His, Thr, Val, Lys, Ile, Leu, Phe, Cys, Gly, Ser, Ala, Pro, and Tyr) were also reduced by approximately 5.5%, in the study of Soleimani et al. (2010). Regarding other nutrients, none of these studies observed an impact on crude fat digestibility, and only Kim et al. (2020) measured a reduction in NDF digestibility with laying hens.

Several mechanisms have been proposed to explain possible negative effects of HS on nutrient digestibility. Lower expression and activity of digestive enzymes, including trypsin, chymotrypsin, lipase, amylase, and maltase, have been observed in broilers reared under high temperatures (Hai et al., 2000; Song et al., 2018; Al-Zghoul et al., 2019). As described in Part I, oxidative stress induced by HS aggravates intestinal barrier disorders (Brugaletta et al., 2022), and hyperthermia has been associated with a reduction in upper gastrointestinal tract blood flow that can induce degradation of the intestinal mucosa (Song et al., 2014; Chegini et al., 2018). Following hot temperature exposure, the absorptive surface area of the small intestine is decreased due to a reduction in villi height, crypt depth (Song et al., 2018; He et al., 2019), and relative jejunal weight (Garriga et al., 2006). Heat stress also modulates the gene expression of several macronutrient transporters. Expression of glucose transporters SGLT1 and GLUT2 is downregulated when HS persists for several days (Sun et al., 2015; Habashy et al., 2017b; Al-Zghoul et al., 2019; Abdelli et al., 2021; Goel et al., 2021), whereas the expression of GLUT5 for the transport of fructose is increased (Habashy et al., 2017b). Despite the relatively greater decrease in AA digestibility compared to other macronutrients, several studies observed no influence of HS exposure on expression of AA transporters, including CAT1, y+LAT1, PePT1, and r-Bat (Sun et al., 2015; Habashy et al., 2017b; Al-Zghoul et al., 2019). On the other hand, Habashy et al. (2017a) measured a decrease in expression of several AA transporters (i.e., CAT1, LAT1, SNAT1, SNAT 2, SNAT 7, B0AT) after 12 days of HS. However, this reduction was not consistent with the slight increase in AA digestibility ( + 3%) observed in the same study.

Furthermore, even though HS does not seem to markedly affect fat digestibility, several studies have reported decreased intestinal expression of FABP and CD36 which are both involved in the uptake of fatty acids (Sun et al., 2015; Habashy et al., 2017b; Al-Zghoul et al., 2019), whereas the expression of FATP1 was increased under chronic HS (Habashy et al., 2017b).

While the regulation of nutrient transporter gene expression might be directly related to physiological adaptations to HS, it is important to consider that structural damages and the degradation of the epithelium induced by HS might be a potential factor indirectly causing the reduction of intestinal transporters (Habashy et al., 2017b). Overall, the slight decrease and inconsistent results regarding nutrient digestibility seem to indicate that reduced digestibility likely explains only a small proportion of reduced feed efficiency under HS conditions.

## Feeding strategies

Lowering HP and improving heat dissipation are two ways to reduce the adverse effects of HS in poultry. While the reduction of HP is achievable by improving digestibility and by feeding the birds closer to their nutrient and energy requirements, an increased heat dissipation is possible by increasing the amount of water loss by evaporation (Syafwan et al., 2011). Several feeding strategies have been tested to attempt to mitigate the negative impact of hot temperatures through these means.

### Feed restriction and withdrawal

Early studies focused on feed restriction before HS exposure, and its effects on HP and performance. In broiler breeders, feed restriction from 44 to 48 weeks before exposure to 4 days of elevated temperatures resulted in 23% decrease in HP compared with *ad libitum* fed birds. However, fed-restricted birds had a higher HP when adjusted for body weight (BW) differences and expressed per unit of metabolic body size (BW^0.75^). The lower BW of fed-restricted birds was therefore responsible for the reduction in HP and not the feed restriction *per se* (MacLeod and Hocking, 1993). In broilers, no beneficial effect of a preventative feed restriction was measured on performance and carcass quality (Plavnik and Yahav, 1998), but more promising results were obtained when feed restriction was applied during the HS period. Abu-Dieyeh (2006) observed that feed restriction to 75% and 50% of the feed consumption of *ad-libitum* fed broilers reduced rectal temperature, mortality, and feed conversion ratio (FCR). However, feed restriction diminished the rate of BW gain (BWG) and delayed marketing age of the birds.

Similarly, feed withdrawal for at least 6 h during HS decreased the corporal temperature (Yalçin et al., 2001; Özkan et al., 2003; Lozano et al., 2006), mortality (Yalçin et al., 2001) and heterophil-to-lymphocyte ratio (Yalçin et al., 2003) of broilers, indicating a reduction of the adverse effects of HS. Nevertheless, effects on performance were not consistent throughout the studies, with some observing a growth improvement (Yalçin et al., 2001; Mohamed et al., 2019) and others reporting a growth degradation (Lozano et al., 2006) likely due to the timing and magnitude of feed restriction (Özkan et al., 2003). Therefore, a short feed withdrawal during the hottest period of the day appears to be the best strategy to minimize the negative effects of HS on growth and delayed market ages. Removing the feed a few hours before the HS period could also be beneficial to avoid the potential increased in HP induced by anticipatory feeding behavior observed in birds exposed to repeated intermittent fasting (Fondevila et al., 2020).

### Dual feeding

Dual feeding is characterized by the distribution of two different diets, one more concentrated in protein and the other more concentrated in energy, that are provided either simultaneously for self-selection or in sequential order. Dietary proteins are known to have a higher thermogenic effect compared with carbohydrates (Geraert, 1991), and feeding high protein diets during the coolest period of the day has been hypothesized to improve the thermotolerance of birds. Sequential feeding of high energy and high protein diets decreased body temperature (De Basilio et al., 2001; Lozano et al., 2006) and mortality (De Basilio et al., 2001), but reduced or did not improve the growth of broilers. Syafwan et al. (2012) tested self-selection under hot temperatures by providing a high-protein diet (CP: 299 g/kg; ME: 2,780 kcal/kg) and a high-energy diet (CP: 150.7 g/kg; ME: 3,241 kcal/kg) and showed that choice-fed and control-fed birds with a standard diet (CP: 215 g/kg; ME: 2,895 kcal/kg) performed similarly, although the former had 14% lower protein intake and 6.4% higher energy intake. However, no data on carcass composition were reported, and a lower protein intake could reduce muscle deposition. While a dual-feeding approach might be feasible in tropical areas and less-intensive production systems, Iyasere et al. (2021) estimated that it is not suitable for most commercial production operations due to cost and logistical constraints.

### Wet feeding

Water is the most important nutrient in broiler nutrition, and it plays an essential role for thermoregulation under hot temperatures. Heat stress increases water loss through the respiratory tract as birds pant to increase heat loss by evaporative cooling (Richards, 1970; Bruno et al., 2011). In the light of the importance of water for the nutrition and physiology of broilers, wet feeding attempts to maximize water intake and utilization. Several studies have investigated the effect of wet feeding, i.e., the use of high moisture diets, on poultry performance under thermoneutral conditions (Moritz et al., 2001; Shariatmadari and Forbes, 2005; Khoa, 2007) and during HS. In heat-stressed broilers, Kutlu (2001) measured increased BWG, DM intake, carcass weight, protein content, but also increased abdominal fat and lipid content per unit of carcass weight, and reduced DM conversion efficiency (DM intake/BWG), when feed was mixed with the same amount of water. Similarly, Awojobi et al. (2009) and Dei and Bumbie (2011) observed increased BWG with wet-fed birds (addition from 1 to 2 parts of water to 1 part of dry feed) reared in tropical conditions. In laying hens, Tadtiyanant et al. (1991) reported that wet feeding increased DM intake, but no beneficial effects were found on performance. In contrast to these results, egg production and egg weight were increased by wet feeding in Japanese quails (Okan et al., 1996a; 1996b). Despite somewhat positive impacts of wet feeding in poultry, its application in the field remains limited due to an increased risk of fungal growth and resulting mycotoxicosis in birds (Wasti et al., 2020).

### Feed form (mash vs. crumble vs. pellets) and feed structure (particle size)

Three different forms of feed are generally used in the poultry industry: mash, crumble, and pellets. Under thermoneutral conditions, pelleted feed is known to increase FI and BWG and improve digestibility (Massuquetto et al., 2018; Massuquetto et al., 2019). During summer, increased feed efficiency and egg production of laying hens have been observed for pelleted diets compared with mash diets (Almirall et al., 1997). In broilers exposed to cyclic HS, Cardoso et al. (2022) measured increased FI ( + 10%), BWG ( + 8.3%), CP digestibility ( + 2.3%), and energy utilization (apparent metabolizable energy, AME and nitrogen-corrected apparent metabolizable energy, AMEn) when feeding a pelleted diet compared with a mash diet. However, pelleting did not improve FCR, livability, or the feed production cost to kg of bird produced ratio. Likewise, Hosseini and Afshar (2017a) observed beneficial effects of pelleting on performance and digestibility when comparing mash, crumbled and pelleted diets under similar cyclic HS conditions. These authors also reported improved carcass weight and yield in heat-stressed broilers fed pelleted diets. Comparable performance improvements were obtained by feeding pelleted diets under thermoneutral and HS conditions (Serrano et al., 2013), so it is likely that mechanisms responsible for the positive effects of pelleting under thermoneutrality can be applied to HS conditions. Pelleting feed has been shown to lower feed wastage (Gadzirayi et al., 2006) and increase feed consumption, while concomitantly reducing physical activity and HP (Skinner-Noble et al., 2005; Latshaw and Moritz, 2009). Furthermore, as observed under thermoneutral (Abdollahi et al., 2011; Serrano et al., 2013) and HS conditions (Hosseini and Afshar, 2017b), pelleted diets reduce the relative weight of the digestive tract compared with birds fed mash diets. The pelleting process can further reduce ingredient particle size, reducing the mechanical stimulation of the gizzard and could therefore lower the energy requirements for maintenance. It also could release some inaccessible nutrients and enhance energy utilization, which could explain the increase in abdominal fat observed by Hosseini and Afshar (2017a) with pelleted diets fed under cyclic HS. Other potential benefits of feeding pelleted feeds during HS shown by these authors include increased villus length and villus to crypt depth ratio in the jejunum (Hosseini and Afshar, 2017b) as well as decreased breast HSP70 mRNA expression, breast creatine kinase protein level, and heterophil-to-lymphocyte ratio (Hosseini and Afshar, 2017a). Collectively, these reports indicate that pelleting attenuates the harmful effects of high ambient temperature in broiler chickens.

Concerning particle size, the use of coarse particles (2,280 µm) of corn increased panting compared to finer particles (605 µm) in broilers fed a mash diet under natural HS conditions (Santos et al., 2019). Similar results were found in laying hens under a semiarid environment, where coarser corn particles increased rectal temperature, respiratory rate, and decreased eggshell quality (De Souza et al., 2015). However, while coarse particles may increase the thermal challenge, they are also known to increase FI and improve performance in broilers under thermoneutral conditions (Amerah et al., 2008; Naderinejad et al., 2016). Thus, more research on broiler performance would be required to fully understand the role of ingredient particle size during HS.

## Dietary energy density and lipid supplementation

The marked decrease in FI and in turn, energy intake, caused by elevated temperatures negatively affects bird performance. The effect of HS on energy utilization of feedstuffs, which is usually represented as AME, is still not well defined. Indeed, responses probably depend on the parameters of the HS imposed and characteristics of the diet, as some studies observed an increase in AME due to hot temperatures (Keshavarz and Fuller, 1980; Geraert et al., 1992), some observed no difference between thermoneutral and HS conditions (Yamazaki and Zi-Yi, 1982; Faria Filho et al., 2007; De Souza et al., 2016), and some have reported a decrease in AME with HS birds (Bonnet et al., 1997). However, three studies using the comparative slaughter technique with broilers placed under thermoneutral and HS conditions from d 28 to 42 (Geraert et al., 1996), or d 21 to 42 (Faria Filho et al., 2007; De Souza et al., 2016), indicate a decrease in retained energy and increase in HP per unit of feed when birds are placed under hot temperatures. Similarly, a quadratic effect of the temperature on the energy requirement for maintenance functions was measured by Sakomura et al. (2005), with the lowest requirements estimated at 25.2°C: ME_m_ = BW^0.75^ x (307.87 + 15.63 T + 0.31 T^2^), with T being the temperature (°C) and BW^0.75^ the metabolic body size. Therefore, the relative contribution of maintenance energy requirements to total energy requirements is partly increased by the lower growth of HS birds, but also directly impacted by the increased temperature, which results in a diminishing effect on feed efficiency.

To compensate for lower energy intake of birds during HS, it has become common for producers in hot climate areas to feed higher energy diets (Wasti et al., 2020). Early studies suggested that high dietary energy concentrations could improve bird performance under constant (Dale and Fuller, 1979) and cyclic HS (Dale and Fuller, 1980), but it should be noted that the CP content of the diets were adjusted to energy levels and thus higher in high energy diets. Nonetheless, more recent studies using isonitrogenous diets have confirmed previous observations and showed that an increase in dietary metabolizable energy (ME) between 100 and 200 kcal/kg for broilers improved BWG up to 17% and FCR up to 10% (Raju et al., 2004; Ghazalah et al., 2008; Attia et al., 2011, 2018; Attia and Hassan, 2017) when reared under hot conditions. In addition, decreased skin and rectal temperatures have been observed in HS poultry fed diets with increased ME content (Al-Harthi et al., 2002; Attia et al., 2011). Increasing dietary ME content also improved ready-to-cook yield (Raju et al., 2004), although no improvement in carcass yield was observed by Ghazalah et al. (2008). However, both research groups reported an increased abdominal fat yield, thus the risk of increasing carcass yield from lipid and not protein deposition is a potential disadvantage of increasing dietary ME in HS broilers.

Increasing ME density in the diet is usually achieved by increasing the concentration of added lipid, and this strategy presents several potential advantages for HS birds. Feeding isocaloric diets with either higher proportions of carbohydrates or fat under HS conditions revealed that broilers had better performance when diets were supplemented with poultry fat, palm oil, or soybean oil compared to no fat supplementation (Zulkifli et al., 2006; Ghazalah et al., 2008). These observations are likely explained by the lower heat increment of fat oxidation compared with carbohydrates and proteins. Indeed, as measured by Fuller and Rendon (1977), high fat diets lead to lower heat increment than low-fat diets. Moreover, lipid inclusion improves nutrient digestion by slowing rate of passage (Mateos et al., 1982) and increasing the energy value of other nutrients (Aardsma et al., 2017). Lipid metabolism also generates more metabolic water than carbohydrate and protein catabolism, which can in turn be used for heat dissipation by evaporation (Barboza et al., 2009). Thus, as suggested by Ghazalah et al. (2008), a potential dietary recommendation for broilers exposed to hot temperatures could be to increase the ME level up to 3,300 kcal/kg, with lipid inclusion up to 5%, especially during the finishing period when birds are the most sensitive to high temperatures.

Although increasing dietary lipid additions has been shown to be a promising way to increase bird performance under HS conditions, less research has been conducted to compare the efficacy of different lipid sources. Zulkifli et al. (2006) did not observe a difference in BWG and FCR among broilers exposed to 34°C and supplemented either with 8% of palm oil or 8% of soybean oil. Abdominal fat and breast intramuscular fat deposition were also unaffected by the fat source. However, in broilers exposed to HS from 32 to 42 days post-hatch and fed isocaloric diets, improved FCR and BWG were observed when feeding diets with coconut oil or beef tallow than with diets containing olive or soybean oil (Seifi et al., 2020). The fatty acids within coconut oil and tallow are rich in saturated fatty acids and have chain lengths of mainly 12 and 16 carbons, respectively, while olive oil and soybean oil are rich in unsaturated fatty acids and have predominantly 18 carbon fatty acids. Short and medium chain fatty acids (SCFAs/MCFAs), containing up to 12 carbon atoms, are absorbed and metabolized more rapidly than longer chains, as they are transported to the portal vein as free fatty acid and do not require any transporter to get absorbed (Guillot et al., 1993), which could reduce the HP induced by digestion. Recent research also suggests saturated fatty acids, SCFAs, and MCFAs could have a beneficial impact on the mitochondrial metabolism and electron transport chain (Schönfeld and Wojtczak, 2016; Seifi et al., 2018, 2020; Hecker et al., 2021), which are known to be disrupted under HS condition (Akbarian et al., 2016).

## Influence of dietary crude protein content

**FIGURE 1:**
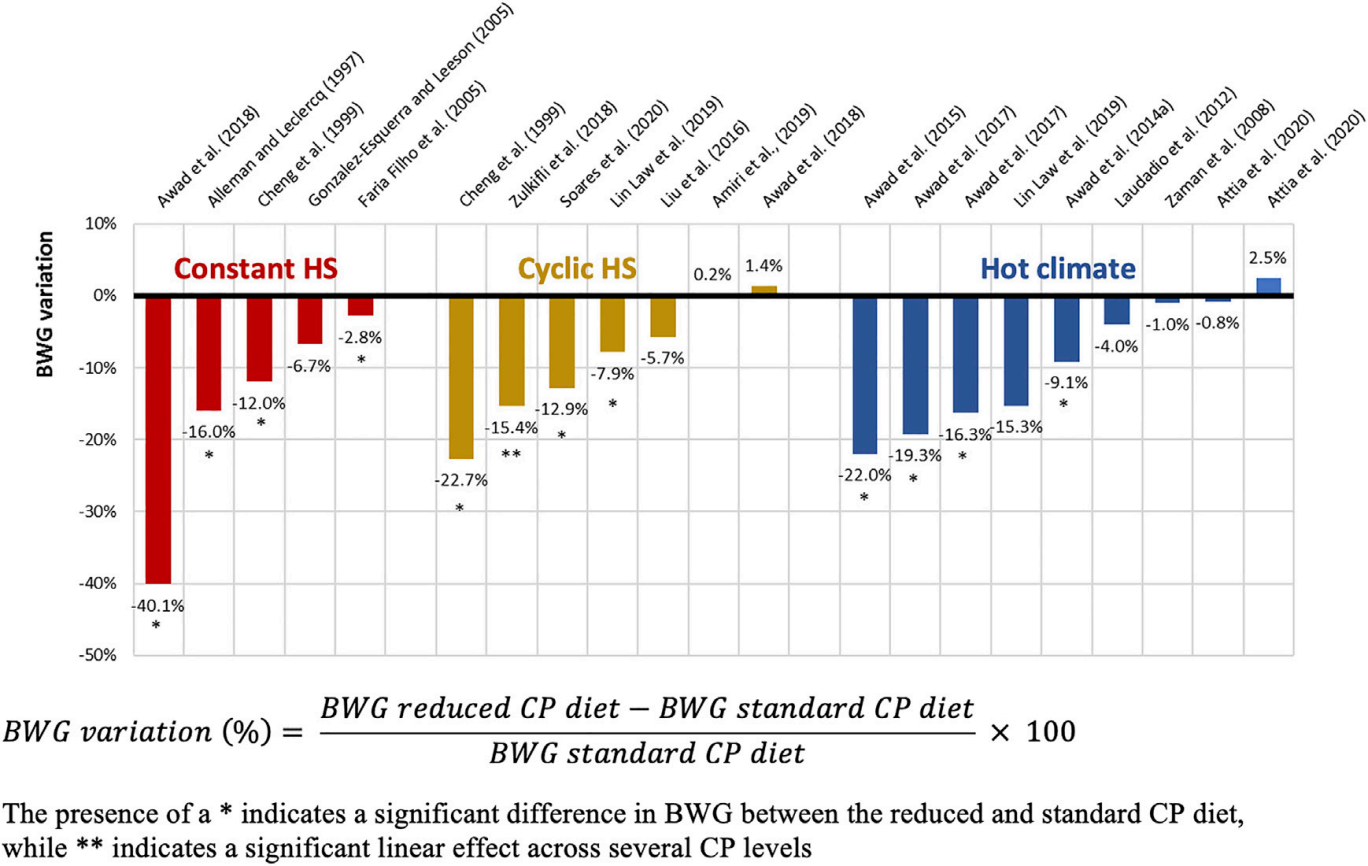
Effect of reduced and standard CP diets on BWG of broilers exposed to different HS conditions.

**FIGURE 2:**
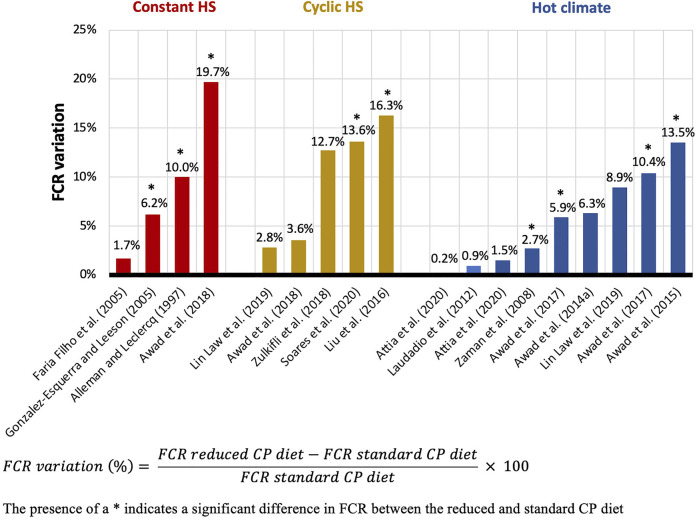
Effect of reduced and standard CP diets on FCR of broilers exposed to different HS conditions.

Proteins have a higher caloric increment than carbohydrates and fat (Musharaf and Latshaw, 1999) and therefore increase the diet-induced HP. When AA are metabolized for energy by birds, much of the HP is caused by deamination reactions and incorporation of N into uric acid (Smith et al., 1978; Swick et al., 2013). Therefore, optimizing dietary CP composition to better fit bird requirements decreases the heat produced during AA oxidation. So, in an effort to reduce the energy released during digestion, absorption, and metabolism of nutrients, dietary CP reductions have been proposed as a strategy to mitigate the harmful effects of HS in poultry (Furlan et al., 2004). Numerous studies in broilers have tested the effects of feeding a reduced CP diet versus a standard CP diet under constant HS (Alleman and Leclercq, 1997; Cheng et al., 1999; Faria Filho et al., 2005; Gonzalez-Esquerra and Leeson, 2005; Awad et al., 2018), cyclic HS (Cheng et al., 1999; Liu et al., 2016; Awad et al., 2018; Zulkifli et al., 2018; Amiri et al., 2019; Lin Law et al., 2019; Soares et al., 2020) and hot climates (Zaman et al., 2008; Laudadio et al., 2012; Awad et al., 2014, 2015, 2017; Lin Law et al., 2019; Attia et al., 2020). Table 1 summarizes 21 HS broiler trials comparing reduced CP diets (ranging from 143 to 190 g/kg CP) and standard CP diets (ranging from 183 to 223 g/kg CP), with both diets in each study formulated to meet or exceed a specific nutritional requirement, such as the Nutrient Requirements of Poultry (NRC, 1994) or breeder recommendations, or to contain similar AA profiles. Approximately half of these studies observed a significant reduction in performance when feeding broilers the reduced CP diet compared to the standard CP diet, while the other half did not observe dietary effects. The response variability can be partly explained by the range of low and standard CP levels, as well as the intensity and duration of the HS period, but it is important to note that feeding a low CP diet without degrading performance is still beneficial for reducing nitrogen excretion. Results for BWG, presented in Figure 1, indicate that regardless of the HS challenge type, reduced CP diets decreased BWG by 10.8% on average (ranging from a reduction of 40.1% to an improvement of 2.5%). Similar results were obtained with FCR, with an average increase of 6.9% (ranging from a decrease of 0.9% to an increase of 19.7%) when dietary CP was reduced (Figure 2). Some studies also reported a decreased FI with reduced CP diets (Cheng et al., 1999; Awad et al., 2014, 2015, 2017, 2018). In addition to a reduced CP diet, some researchers tested the effects of a higher CP diet, with CP levels above the standard recommendations. During HS, high CP diets resulted in a decrease (Cheng et al., 1999) or an increase in BWG (Faria Filho et al., 2005) and a decrease in FCR (Cheng et al., 1999; Faria Filho et al., 2005; Gonzalez-Esquerra and Leeson, 2005). However, other studies reported no effect of high versus standard CP diets (Zaman et al., 2008; Laudadio et al., 2012; Soares et al., 2020) and the increased diet cost associated with high CP diets could result in detrimental economical scenarios (Cardoso et al., 2022).

**TABLE 1:** Summary of experimental conditions of broiler studies comparing reduced and standard CP diets under HS conditions.

Heat stress condition	Heat stress length	Average temperature (°C)	Standard CP (g/kg)	Reduced CP (g/kg)	Age start (d)	Age end (d)	Duration (d)	References
Constant HS	—	34	194	143	22	42	20	Awad et al. (2018)
	—	32	199	160	23	44	21	Alleman and Leclercq (1997)
	—	32.2	198	161	21	49	28	Cheng et al. (1999)
	—	31.4	200	180	21	42	21	Gonzalez-Esquerra and Leeson (2005)
	—	33	200	185	7	21	14	Faria Filho et al. (2005)
Cyclic HS	35°C for 8 h	29.4	198	161	21	49	28	Cheng et al. (1999)
	33°C for 6 h	25.5	190	162	22	35	13	Zulkifli et al. (2018)
	32°C for 8 h	26	200	160	22	42	20	Soares et al. (2020)
	34°C for 7 h	26.2	183	167	22	42	20	Lin Law et al. (2019)
	—	27.8	213	153	28	42	14	Liu et al. (2016)
	34°C for 8 h	NA	195	175	0	42	42	Amiri et al. (2019)
	34°C for 7 h	26.2	194	143	22	42	20	Awad et al. (2018)
Hot climate	—	At least 28.1	223	161	0	21	21	Awad et al. (2015)
	—	At least 28.1	223	162	0	21	21	Awad et al. (2017)
	—	At least 28.3	223 and 194	162 and 135	0	42	42	Awad et al. (2017)
	—	At least 28.5	216 and 187	176 and 156	0	35	35	Lin Law et al. (2019)
	—	At least 28.3	207	177	0	21	21	Awad et al. (2014)
	—	NA	205	185	14	42	28	Laudadio et al. (2012)
	—	NA	210	190	0	28	28	Zaman et al. (2008)
	—	34	190	155	28	49	21	Attia et al. (2020)
	—	35	186	152	30	45	15	Attia et al. (2020)

Feed-grade AA, which are included at higher levels in reduced CP diets to meet digestible AA requirements, allow to provide a balanced AA diet, and minimize the HP caused by AA oxidation, which is not possible to reach when relying on feed sources only. They also do not need enzymes for digestion and, as such, do not contribute to the digestion-related production of body heat (Morales et al., 2020). However, the performance degradations reported with reduced CP diets aligns with the lower HP observed in birds fed a high CP diet (220 g/kg) versus a low CP diet (160 g/kg) under cyclic HS conditions (Soares et al., 2020). Similar results have also been obtained under constant HS when comparing a high (230 g/kg), standard (200 g/kg), and low (170 g/kg) CP diets (Faria Filho et al., 2007). The lack of interaction between the CP level and environmental temperature reported by these authors is supported by studies conducted under thermoneutral conditions, where no difference (Noblet et al., 2003, 2007) or an increase (Swennen et al., 2004) in HP was measured with low CP diets, indicating that HS is not the cause *per se* of the higher HP with reduced CP diets. These results are surprising due to the higher caloric increment of proteins, but a possible explanation is that standard CP diets are usually formulated with a higher oil inclusion rate to reach the same amount of energy than reduced CP diets which generally have higher inclusion of corn (Soares et al., 2020). The extra-metabolic effect of dietary lipids, where the metabolizable energy value of the lipid exceeds its gross energy value (Aardsma et al., 2017), could compensate for the possible increase in heat increment derived from protein (Soares et al., 2020). Interestingly, reduced CP diets with AA deficiencies have also been associated with a greater plasma level of triiodothyronine (Carew et al., 1983, 1997; Buyse et al., 1992), which is known for its thermogenic effect (Collin et al., 2003).

Overall, simultaneously increasing dietary energy and CP could be a potentially beneficial strategy to limit the adverse effects of HS on broiler growth and feed efficiency. Indeed, improved performance has been demonstrated under HS conditions when broilers were fed both a high dietary ME and CP contents (Attia et al., 2006; Attia and Hassan, 2017). However, in a similar study in which broilers were exposed to thermoneutral temperatures or cyclic HS from day 19–42 and were fed with a dietary ME and CP content of 3,152 kcal/kg and 194.8 g/kg or 3,253 kcal/kg and 210.3 g/kg, respectively, no improvement in performance was observed with the higher nutrient and energy density diet in either environment. Consequently, an economic evaluation actually showed a decrease in overall profitability with the higher density diets (Cardoso et al., 2022).

The conflicting evidence of higher caloric increment of dietary protein and impaired performance of broilers fed reduced CP diets led Gonzalez-Esquerra and Leeson (2006) to conclude that no consensus has been reached on protein requirements of heat-stressed birds. More recent trials on reduced CP diets have shown no performance improvements or amelioration of HP reduction and do not support this dietary strategy under HS conditions. Nonetheless, when following the “ideal protein” concept, where all essential digestible AA are provided in balance (Baker and Chung, 1992), the supplementation of unbound feed-grade AA in reduced CP diets to satisfy the bird’s requirements should result in similar performance as when feeding standard CP diets. Furthermore, most of the studies presented above based their requirements on NRC or breeder recommendations, albeit broiler’s AA requirements under HS conditions still remain undefined. More importantly, although those studies met specific nutritional requirements for essential AA, some potentially limiting AA such as Arg, Thr, Ile, Leu, His, and Phe were not equally balanced between diets. Diets with AA imbalance can lead to adverse effects especially under HS condition as they normally increase HP (Sekiz et al., 1975). Also, the FI reduction triggered by HS reduces the amount of CP and AA ingested by the birds, potentially resulting in deficiency when compared with reduced dietary concentrations. Therefore, even if the inclusion level for all AA was formulated to meet or exceed a target nutritional requirement under thermoneutral conditions, the effective AA consumption may not have reached the bird’s requirements for some AA under HS conditions.

Further research on dietary CP and its interaction with energy and AA content would be required to better characterize the biological response induced by those diet changes under HS conditions. This would allow for a better understanding on the utilization of those nutrients in poultry reared under hot temperature to ultimately facilitate better prediction of economic outcomes associated with nutritional dietary variation.

## Supplementation of amino acids

### Amino acid density

Altering dietary density of essential AA has been shown to have promising results in heat-stressed broilers. In broilers under hot temperatures, Maharjan et al. (2020) fed five levels of digestible Lys (dLys) from 80% to 120% of the recommended level with all other AA:dLys ratios held constant and observed quadratic responses in average daily gain and FCR up to the 120% dLys level, and no influence on FI. In contrast, the optimal average daily gain and FCR were closer to the 100% recommendation of dLys under thermoneutral conditions. This indicates a potential increase in overall AA requirements under HS, although the authors concluded that the requirement of AA/Mcal was not different in hot or thermoneutral environments, which was also observed by Hruby et al. (1995). Moreover, Alhotan et al. (2021) fed an AA density ranging from 80% to 110% of breeder recommendations to broilers exposed to cyclic HS. In contrast with the results reported by Maharjan et al. (2020), no interactions between environmental temperature and dietary AA density were observed on performance and processing data. However, linear effects of AA density indicated that BWG, feed efficiency, and breast muscle yield responded to increased AA density in both environments. Even though FCR was numerically improved by 10 points with the 110% AA diet relative to the 100% AA diet, this difference was not statistically significant and may indicate that higher AA levels were above the bird’s requirements. In another trial, increasing the density of Met, Lys, and Thr in a reduced CP diet increased production performance of cyclically heat-stressed broilers over the ones obtained with standard CP diet and, in addition, improved intestinal health as indicated by changes in small intestinal morphology and increased mRNA expression of some tight junction proteins (Wang et al., 2022). Therefore, increasing AA density could be beneficial for broilers experiencing HS, especially when achieved with free AA to minimize diet-induced thermogenesis. However, further research is required to better characterize the true AA requirements of birds under HS conditions.

### Individual amino acid supplementation

Methionine (Met) is the first limiting AA in avian species and is considered, along with cysteine (Cys), to meet total sulfur AA (TSAA) needs for the bird. Because of its importance in maintenance functions and muscle deposition that are greatly impacted during exposure to HS, defining Met requirements is an important step in optimizing poultry nutrition under HS conditions. Indeed, higher requirements of Met have been found in broilers under high temperatures compared to thermoneutral conditions (Silva Junior et al., 2006; Sahebi-Ala et al., 2021), but this does not appear to be the case in laying hens or pullets (Bunchasak and Silapasorn, 2005; Castro et al., 2019). Several physiological mechanisms have been proposed regarding the importance of Met under HS. First, Met supplementation has been shown to increase the antioxidant capacity of broilers (Del Vesco et al., 2015b; Gasparino et al., 2018; Liu et al., 2019; Santana et al., 2021). Under thermoneutral conditions, the production of reactive oxygen species and the antioxidant systems in chickens are balanced and can adapt to overcome normal challenge. Acute and chronic HS disturb this equilibrium due to an overproduction of reactive oxygen species, which ultimately surpasses the antioxidant capacity and leads to oxidative stress (Lin et al., 2000, 2006a; Azad et al., 2010; Akbarian et al., 2016). Furthermore, Met supplementation affected the inflammation-related gene expression in the liver of broilers placed under high temperature (Liu et al., 2019). Another potential benefit of Met supplementation under HS is its stimulatory effect on protein deposition and inhibition of protein breakdown as indicated by the increased expression of protein synthesis-related genes IGF1, GHR and PI3KR1 in the liver, and decreased expression of protein degradation-related genes atrogin1 and CTSL2 in the breast (Del Vesco et al., 2013; 2015a).

Beneficial effects of increasing the dietary amount of essential AA other than Met are not as well defined. Dietary levels of Lys, the second limiting AA in broiler chicken diets based on corn and soybean meal (Ishii et al., 2019), are closely associated with muscle protein deposition. However, the growth depression under HS does not seem to be ameliorated by supplementing broiler diets with Lys above the thermoneutral requirements (Mendes et al., 1997; Corzo et al., 2003; Attia et al., 2011). Interestingly, when Lys was supplemented in combination with Met in a reduced CP diet, broilers had similar performance and carcass characteristics to those fed a higher CP diet under hot climate conditions (Attia et al., 2020). In this study, additional treatments with supplementation of other essential AA besides Met and Lys did not ameliorate performance reductions caused by HS, emphasizing the potential importance of those two AA under HS conditions.

Threonine is almost invariably the third limiting AA in poultry diets (Kidd, 2000). In broilers, the earliest studies on Thr supplementation above the estimated requirements for birds under hot temperatures showed no or minimal benefits on performance (Dozier et al., 2000; Kidd et al., 2000; Ojano-Dirain and Waldroup, 2002; Shan et al., 2003), whereas more recent studies have shown some performance improvements (Debnath et al., 2019; Miah et al., 2022). In laying hens, increasing the supplementation of dietary Thr to 0.66% instead of 0.43% did not improve performance outcomes, but it decreased HSP70 in the ileum (Azzam et al., 2019) and increased SOD concentration in both serum and liver (Azzam et al., 2012), indicating potential antioxidant effects of Thr under HS condition.

Unlike mammals, poultry are highly dependent on dietary Arg supply because of less active *de novo* Arg synthesis pathways in birds (Klose et al., 1938; Tamir and Ratner, 1963; Castro and Kim, 2020). In broilers, the determination of Arg requirements under HS conditions have led to inconsistent results among different age periods. Over-supplementation was detrimental from 1 to 3 weeks of age (Chamruspollert et al., 2004), neutral from 3 to 6 weeks of age (Mendes et al., 1997), and beneficial from 6 to 8 weeks of age (Brake, 1998). Arg supplementation also improved FCR of Pekin ducks exposed to cyclic HS (Zhu et al., 2014) and enhanced several welfare indicators and decreased corticosterone plasma concentration in laying hens during the hot summer period (Bozakova et al., 2015). Furthermore, increasing dietary Arg improved performance, reproduction, antioxidant status, immunity, and maternal antibody transmission in quails (Kalvandi et al., 2022). The ability of Arg to reduce physiological stress is likely to be attributed to its antioxidative effects (Gupta et al., 2005). Arg is also the only nitrogen donor in the production of nitric oxide, which is involved in vasodilatation to potentially aid thermoregulation of heat-stressed birds (Uyanga et al., 2021). Interestingly, more focus is being placed on the potential beneficial effects of citrulline (Cit), a compound synthetized during Arg catabolism and the formation of nitric oxide. Recent studies have shown that Cit supplementation can effectively increase systemic Arg levels, even more than direct L-Arg supplementation (Morita et al., 2014; Agarwal et al., 2017). Cit concentration in blood has also been shown to be modulated by hot temperatures (Chowdhury et al., 2014; Chowdhury, 2019) and its supplementation may increase nitric oxide synthesis, provide an anti-inflammatory response, and enhance the central regulation of body temperature (Chowdhury et al., 2017; Uyanga et al., 2021, 2022).

Leu, Ile, and Val are three essential AA collectively known as branched-chain AA (BCAA). Their roles are diverse and include effects on performance, immunity, and intestinal health. They also serve as signaling molecules in the regulation of glucose, lipid, and protein synthesis (Kim et al., 2022). Kop-Bozbay et al. (2021) investigated the effect of increased BCAA density under HS conditions and did not observe any improvement in growth performance. These authors also tested various dietary Val concentrations and did not observe effects on performance. However, high incorporation of Leu in those diets might have triggered the antagonist effect among BCAA (Ospina-Rojas et al., 2020). Interestingly, in ovo Leu injection improved BWG and thermotolerance of birds during subsequent exposure to HS (Han et al., 2017, 2019, 2020; Chowdhury et al., 2021). With the current increasing availability of feed-grade Val and Ile, further work is needed to define the potential for BCAA to combat HS in poultry.

Trp is an essential AA in poultry diets due to its need for protein synthesis, as well as serotonin and niacin production (Le Floc’h et al., 2011). Few studies have been published on the requirements of Trp under HS conditions, although high dietary concentrations did not improve performance in broilers (Tabiri et al., 2002; Shan et al., 2003; Badakhshan et al., 2021) or layers (Dong et al., 2012). However, Trp supplementation did decrease rectal temperature and abated corticosterone responses caused by HS in broilers (Badakhshan et al., 2021). Trp supplementation also increased eggshell quality and decreased SOD serum concentration in laying hens during HS (Dong et al., 2012). To our knowledge, no studies on the effect of dietary supplementation of less-limiting essential AA beyond Trp, such as His and Phe, have been conducted in poultry subjected to HS.

The remaining AA are non-essential AA and can be synthesized from other precursors. Besides altering essential AA needs, the reduced FI caused by HS limits the amount of nitrogen consumed by birds, which could potentially lead to a lack of sufficient nitrogen quantity for non-essential AA synthesis (Awad et al., 2014). Feeding low CP diets during hot temperatures could also worsen this nitrogen deficiency. Birds fed a diet with increased essential and non-essential AA concentrations under HS had a better performance than when a diet with only increased essential AA concentrations was fed (Awad et al., 2014, 2015). However, when comparing individual supplementation of several non-essential AA in low CP diets, only Gly improved broiler FCR under both normal and acute HS conditions (Awad et al., 2015, 2018). Recent research also suggests that Gly and Ser, which are normally evaluated together as Gly equivalents, are co-limiting or limiting before some BCAA in low CP diets under thermoneutral conditions (Chrystal et al., 2020; Maynard et al., 2022), which could make Gly equivalents important AA to consider during reduced FI caused by HS.

Therefore, for the essential AA, it seems possible that supplementation of Met and potentially Arg above current requirements could be beneficial under HS condition. However, further research is required to elucidate the effects of other essential AA, as well as Gly, non-essential AA, and overall nitrogen supply.

## Conclusion

**FIGURE 3:**
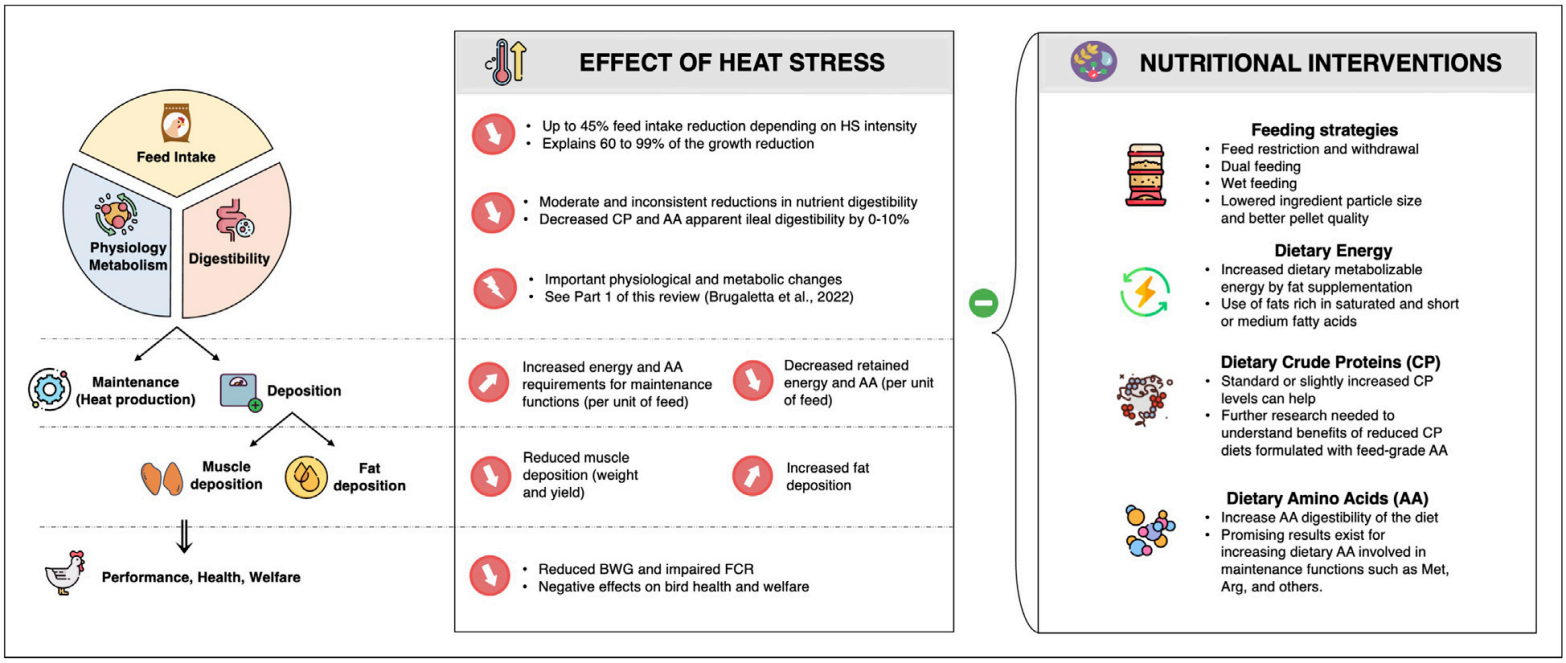
Conclusive scheme of the beneficial nutritional interventions on broilers exposed to heat stress conditions.

Adaptating to rising global temperatures while maintaining production efficiency is an important emerging challenge for the poultry industry. Under hot temperatures, birds reduce their FI to lower HP, and this is the main factor explaining the degradation of bird performance (Figure 3). Mitigation of those negative effects requires a holistic approach, and adjusting feeding practices and nutritional programs have a critical role to play. Even though some feeding strategies are difficult to implement in the field, especially with intensive rearing systems, several practices discussed in this review have shown beneficial effects to reduce the heat load on poultry. Increasing dietary lipid concentration and maintaining a standard CP level are also recommended to compensate for the FI reduction and better fit the birds’ requirements under elevated temperatures. Considering an increase in the density of some AA, like methionine and arginine, to meet the increased AA requirements for maintenance functions could also be advantageous. Therefore, further research is required to characterize nutrient partitioning and requirements of birds under HS conditions to ensure efficient and cost-effective solutions for the poultry industry.

## Abbreviation

AA, amino acid; AME, apparent metabolizable energy; AMEn, nitrogen-corrected apparent metabolizable energy; BCAA, branched-chain AA; BW, body weight; BWG, body weight gain; Cit, citrulline; CP, crude protein; DM, dry matter; dLys, digestible Lysine; FCR, feed conversion ratio; FI, feed intake; HP, heat production; HS, heat stress; ME, metabolizable energy; TSAA, total sulfur AA.
